# Effect of Hepatitis B virus infection during pregnancy on the risk of postpartum hemorrhage: a systematic review and meta-analysis

**DOI:** 10.3389/fgwh.2025.1596520

**Published:** 2026-01-09

**Authors:** Jingwen Chen, Ke Deng, Peng Zhao, Mingyu Liao, Jin Guo, Chunrong Liu, Qixin Cai, Kang Zou, Yiquan Xiong, Jing Tan

**Affiliations:** 1 General Practice Medical Center, Chinese Evidence-based Medicine Center, West China Hospital, Sichuan University, Chengdu, Sichuan, China; 2 NMPA Key Laboratory for Real World Data Research and Evaluation in Hainan, Chengdu, China; 3 Sichuan Center of Technology Innovation for Real World Data, Chengdu, China; 4 Acupuncture and Tuina, College of Chinese Medicine, Chongqing Medical University, Chongqing, China

**Keywords:** adverse pregnancy outcome, HBsAg, Hepatitis B virus, postpartum hemorrhage, pregnancy

## Abstract

**Aim:**

Hepatitis B virus (HBV) infection during pregnancy is one of the most common comorbidities, which may increase the risk of adverse obstetric and perinatal outcomes. However, the association between maternal HBV infection and postpartum hemorrhage (PPH) remains uncertain. The aim of this study is to evaluate whether maternal HBV infection will increase the risk of PPH.

**Methods:**

Five English and three Chinese databases were searched from inception to 30 June 2024, with the aim to include recently published eligible studies. Cohort and case–control studies that evaluated the association between maternal HBV infection and PPH were included. The Newcastle–Ottawa scale was used to evaluate the risk of bias for the included studies. We pooled crude relative risk (cRR) and adjusted odds ratio (aOR) as effect sizes. Three subgroup analyses and seven sensitivity analyses were performed.

**Results:**

A total of 21 cohort studies involving 379,782 participants were included. The pooled results of the unadjusted data revealed that maternal HBV infection was associated with an increased risk of PPH [cRR = 1.18, 95% confidence interval (CI): 1.06–1.31]. Furthermore, the pooled results of adjusted data showed a similar risk of PPH (aOR = 1.50, 95% CI: 1.29–1.73). The effect was similar in three subgroup analyses (i.e., sample size, study region, and prevalence of HBV infection). Sensitivity analyses confirmed that the primary results were robust.

**Conclusions:**

Maternal HBV infection is associated with an increased risk of PPH. Further studies are warranted to evaluate the impact of maternal HBeAg serostatus and HBV DNA levels on PPH.

**Systematic Review Registration:**

identifier CRD42023442626.

## 1 Introduction

Hepatitis B virus (HBV) infection is a significant worldwide public health challenge. The global rate of seroprevalence of hepatitis B surface antigen (HBsAg) is estimated at 3.5%, with approximately 296 million people living with chronic HBV infection as of 2019 (1). The burden of HBV infection varies widely in different parts of the world. The Western Pacific and African regions carry the highest burden, with 116 million and 81 million people infected, respectively. In contrast, the burden of HBV infection is lowest in the Americas, with only 5 million people infected (1, 2).

Beyond the general population, maternal HBV infection is common worldwide and is one of the most common pregnancy comorbidities. For example, the rate of prevalence of maternal HBV infection in China has been reported to be 5.49% (3). Several studies have shown that maternal HBV infection is associated with an increased risk of adverse obstetric and perinatal outcomes, including preterm birth (4), gestational diabetes mellitus (GDM) (5), and intrahepatic cholestasis of pregnancy (ICP) (6). However, uncertainty with regard to whether maternal HBV infection is associated with an increased risk of postpartum hemorrhage (PPH), which is one of the most common pregnancy-related complications (7, 8), persists.

Postpartum hemorrhage (PPH) is the leading cause of maternal mortality worldwide, accounting for nearly 25% of all pregnancy-related deaths (9). Several studies have investigated the potential association between maternal HBV infection and PPH, but their findings have been contradictory. For instance, a multicenter retrospective cohort study that included 22,374 participants reported that maternal HBV infection significantly increased the risk of PPH (10). However, several other studies reported no significant association between maternal HBV infection and PPH (11, 12). To address this important clinical question, in this study, we conduct a systematic review and meta-analysis to explore whether maternal HBV infection is associated with an increased risk of PPH.

## 2 Methods

This study was registered with PROSPERO (CRD42023442626) and conducted in accordance with the meta-analysis of observational studies in epidemiology (MOOSE) guidelines (13).

### 2.1 Eligibility criteria

The systematic review question was formulated using the PECO framework, in which the components are defined as follows: P (Population: pregnant women), E (Exposure: maternal HBV infection), C (Comparison: pregnant women without HBV infection), and O (Outcome: PPH). Cohort studies and case–control studies evaluating the association between maternal HBV infection and PPH were eligible for inclusion. We excluded studies in which all pregnant women were diagnosed with HBV infection combined with other diseases (e.g., COVID-19 or ICP) or had received antiviral therapy. Reviews, comments, case reports, letters, and editorials were excluded. When data from the same study were reported in multiple publications, the report with the largest sample size was included.

### 2.2 Literature search

We initially performed a search of eight databases from inception to 26 June 2023 with an update on 30 June 2024. These databases were five English sources (PubMed, Embase, Scopus, Cochrane Library, and Web of Science) and three Chinese databases (China National Knowledge Infrastructure, Wanfang databases, and Weipu databases). Searches in Chinese databases were limited to those journals included in the Peking University Core Periodical Catalog, which represents excellent Chinese journals (14). All searches were conducted without any language limitations. Both Medical Subject Headings (MeSH) and free-text terms—such as “Hepatitis B,” “HBV,” “Postpartum Hemorrhage,” “Pregnancy Outcome,” “Cohort Studies,” and “Case-Control Studies”—were used to develop the search strategy. The detailed search strategy is provided in Supplementary Appendix S1.

### 2.3 Study process and data extraction

The retrieved studies were imported into the Endnote library. After removing duplicates, three researchers (JW, KZ, and MY) independently screened titles and abstracts and assessed full texts using the predefined criteria to select the final eligible studies. Any disagreements were resolved through discussion or, if necessary, mediation by a third party (YQ). Additional relevant studies were identified manually by searching the reference lists of the included studies.

Two researchers (JW and JG) independently extracted data from eligible studies using a standardized and predetermined form, which included the following information: study characteristics (e.g., first author, publication year, publication language, study location, study design, time of data collection, and sample size); included participant characteristics (e.g., maternal age); HBV infection characteristics (e.g., diagnostic criteria of HBV infection, the prevalence of HBV infection); PPH characteristics (e.g., diagnostic criteria of PPH, the incidence of PPH); exposed and unexposed characteristics (e.g., number of HBV infection pregnancies and total pregnancies in each group); and effect estimates (e.g., number of PPH cases in the exposed and control groups, adjusted data, and relevant confounders). Any disagreements were resolved through discussion.

### 2.4 Exposure and outcome definitions

In this study, maternal HBV infection was defined as HBsAg seropositivity during pregnancy, regardless of the hepatitis B e antigen (HBeAg) serostatus. In this study, PPH referred to primary PPH, defined as blood loss of more than 500 mL after vaginal delivery or more than 1,000 mL after cesarean delivery within 24 h of delivery (15). Since there has been a slight change in the definition of PPH in recent years, pregnant women who meet the American College of Obstetricians and Gynecologists criteria are also considered to have PPH; according to that criteria, PPH is defined as a cumulative blood loss of 1,000 mL or more or blood loss associated with signs or symptoms of hypovolemia within 24 h of delivery, regardless of the mode of delivery (16).

### 2.5 Quality assessment

Two researchers (JW and PZ) independently assessed the risk of bias using the Newcastle–Ottawa scale (NOS), which consists of three parameters: (1) selection, (2) comparability, and (3) outcome (17). The NOS includes eight assessment items with a maximum score of 9, with comparability contributing up to 2 points. We categorized the studies as low, moderate, and high quality, with NOS scores of 1–3, 4–6, and 7–9, respectively. Disagreements were resolved through consensus with a third reviewer (YQ).

### 2.6 Statistical analyses

We pooled the crude relative risk (cRR), adjusted odds ratio (aOR), and corresponding 95% confidence intervals (CIs) between HBV infection and PPH using the random-effects model (DerSimonian–Laird). The heterogeneity of effect sizes among included studies was assessed using the *I*^2^ statistic and Cochran's *Q* test (18), with *I*^2^ > 50% indicating statistically significant heterogeneity between studies. We performed three subgroup analyses: (1) sample size (more than 1,000 vs. less than 1,000), (2) study region (Asia vs. non-Asia), and (3) prevalence of HBV infection (higher vs. lower). A study by Schweitzer et al., based on 1,800 HBsAg prevalence reports from 161 countries, defined a higher rate of prevalence of HBV infection as >3.61% (3). Furthermore, studies that did not explicitly report prevalence also referred to the data reported by Schweitzer et al. (3). We used the interaction test to estimate the differences between the subgroups (19). In addition, we performed seven sensitivity analyses: (1) omitting studies with NOS scores of less than 7; (2) omitting studies published in non-English languages; (3) omitting studies published as conference abstracts; (4) omitting studies with HBsAg serologic testing not performed during first trimester; (5) omitting studies including pregnancies that received antiviral therapy; (6) omitting studies without reported diagnostic criteria for PPH; and (7) performing a trim-and-fill analysis to evaluate the robustness of pooled effect estimates (20). Publication bias was assessed using funnel plots and Egger's tests (21). All statistical analyses were performed using Stata 18.0 (StataCorp LLC, College Station, TX, USA).

## 3 Results

**Figure 1:**
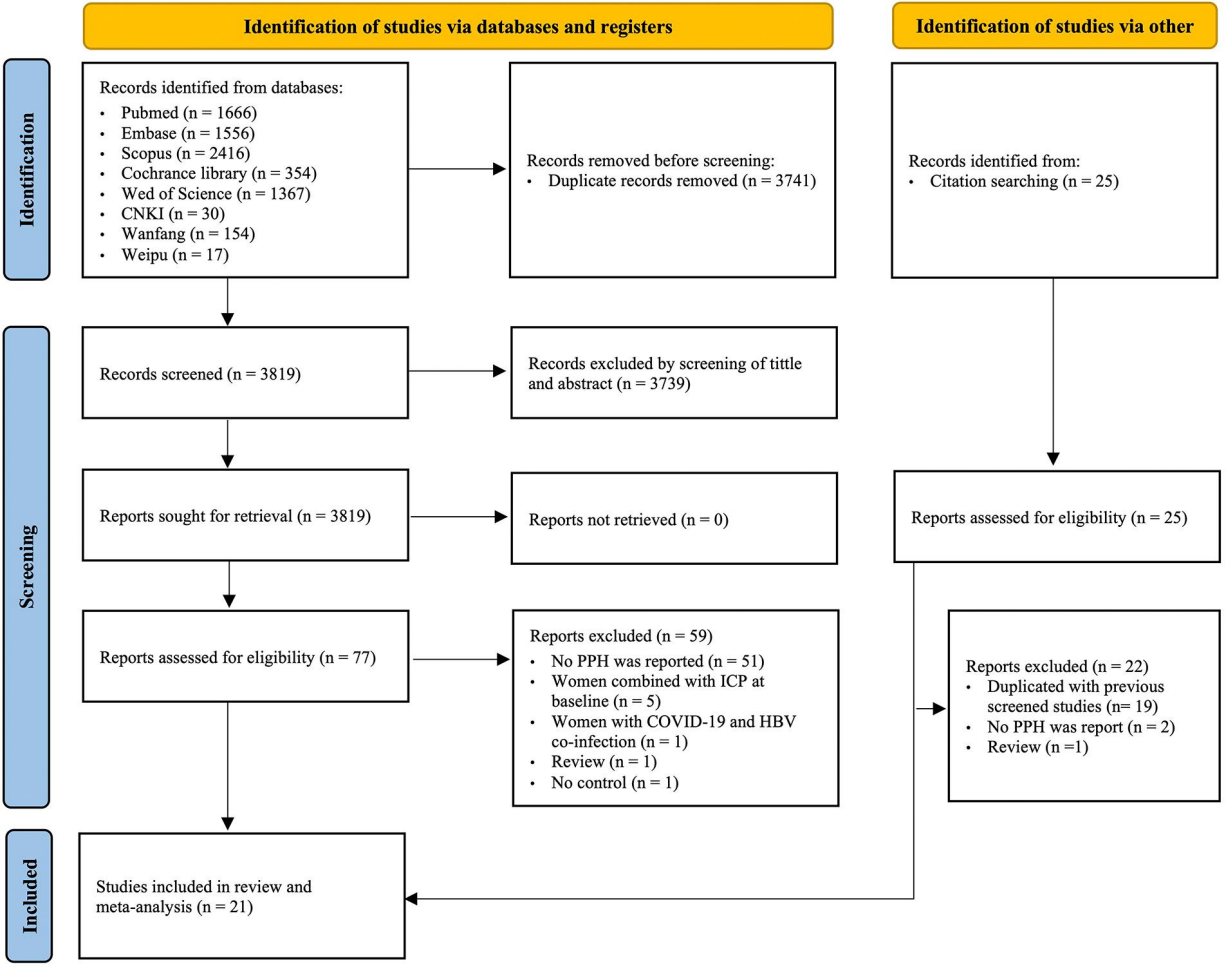
A flowchart of literature search and selection of articles. CNKI, China National Knowledge Infrastructure; PPH, postpartum hemorrhage; ICP, intrahepatic cholestasis of pregnancy; HBV, hepatitis B virus.

A total of 7,560 studies were retrieved in the initial search across multiple databases. After deduplication and title, abstract, and full-text screening, 21 retrospective cohort studies were finally included (7, 8, 10–12, 22–37). A flowchart of the selected literature is presented in Figure 1.

### 3.1 Study characteristics

The characteristics of the included studies are summarized in Table 1. A total of 21 studies enrolling 379,782 participants, with 31,069 HBV-infected pregnant women, were included. Sample sizes ranged from 163 to 87,889, with 12 studies recruiting more than 1,000 pregnant women (7, 8, 10–12, 25–30, 37). These studies were conducted in China (*n* = 15) (7, 8, 10–12, 22–28, 30, 32, 36), Thailand (*n* = 2) (34, 35), Korea (*n* = 1) (37), Iran (*n* = 1) (33), Australia (*n* = 1) (29), and Romania (*n* = 1) (31). Seventeen studies were published in English (7, 8, 10–12, 29–31, 33, 35, 37), three in Chinese (28, 32, 36), and one in Thai (34). Three studies included pregnancies that received antiviral therapy. Of these, two studies specified the number: Lok et al. reported 44 individuals (0.5% of the total HBV-infected pregnancies) and Tu et al. reported 69 individuals (17.4%) (22, 27). However, Chen et al. did not specify the number of individuals who received antiviral therapy (24). Among the included studies, 14 did not specify the diagnostic criteria for PPH (7, 10, 12, 24, 28–37). Of the remaining seven, five provided detailed definitions (11, 22, 23, 25, 27), while two defined the outcome based on ICD criteria (8, 26).

**Table 1:** Characteristics of included studies.

Study	Study region	Study design	Publication language	No. of center	Period of data collection	No. of participants	Exposure group	Prevalence of HBV positivity	Diagnostic criteria of PPH	Multivariable analysis
Tu (22)^a^	China	Retrospective cohort	English	1	2015–2022	794	HBsAg+	Not reported	Vaginal delivery ≥500 mL and cesarean section ≥1,000 mL	Multivariate^c^
Mao (23)	China	Retrospective cohort	English	1	October 2016 to October 2020	994	HBsAg+	Not reported	Blood loss of ≥500 mL for vaginal delivery and ≥1,000 mL for cesarean section	Multivariate^d^
Chen (24)^a^	China	Retrospective cohort	English	1	January 2011 to December 2021	315	HBsAg+	Not reported	Not reported	Matching^e^
Huang (25)	China	Retrospective cohort	English	1	October 2018 to June 2020	3,808	HBsAg+	7.30%	Loss of blood equal to or greater than 500 mL within 24 h after delivery	No
Weng (12)	China	Retrospective cohort	English	1	January 2018 to June 2022	25,114	HBsAg+	8.34%	Not reported	No
Chen (11)	China	Retrospective cohort	English	1	January 2015 to March 2020	19,428	HBsAg+	3.78%	Blood loss of ≥500 mL for vaginal delivery, ≥1,000 mL for cesarean section	No
Yin (26)	China	Retrospective cohort	English	1	January 2009 to December 2019	39,539	HBsAg+	7.70%	ICD-9	No
Sun (8)	China	Retrospective cohort	English	1	January 2005 to December 2017	49,479	HBsAg+	3.30%	ICD-10	No
Lok (27)^a^	Hong Kong	Retrospective cohort	English	1	2000–2019	87,889	HBsAg+	9.56%	Blood loss of ≥500 mL for vaginal delivery and ≥1,000 mL for cesarean section	No
Zhang (7)	China	Retrospective cohort	English	1	July 2009 to December 2018	82,775	HBsAg+	11.39%	Not reported	No
Li (28)	China	Retrospective cohort	Chinese	1	February 2010 to December 2011	6,347	HBsAg+	4.36%	Not reported	Multivariate^f^
Tan (10)	China	Retrospective cohort	English	6	January 2009 to December 2010	22,374	HBsAg+	4.20%	Not reported	Multivariate^g^
Cheng (29)^b^	Australia	Retrospective cohort	English	1	January 2008 to December 2012	23,430	HBsAg+	1.28%	Not reported	Multivariate^h^
Mak (30)	Hong Kong	Retrospective cohort	English	1	October 2010 to December 2011	9,526	HBsAg+	7.85%	Not reported	No
Moga (31)	Romania	Retrospective cohort	English	1	January 2011 to December 2012	163	HBsAg+	Not reported	Not reported	Matching^i^
Lu (32)	China	Retrospective cohort	Chinese	1	May 2009 to July 2011	453	HBsAg+	Not reported	Not reported	No
Saleh-Gargari (33)	Iran	Retrospective cohort	English	1	March 2001 to December 2008	900	HBsAg+	Not reported	Not reported	Matching^j^
Thungsuk (34)	Thailand	Retrospective cohort	Thai	1	January 2005 to December 2007	324	HBsAg+	1.30%	Not reported	Matching^k^
Lert-amornpong (35)	Thailand	Retrospective cohort	English	1	January 2003 to December 2005	326	HBsAg+	1.93%	Not reported	Matching^l^
Xue (36)	China	Retrospective cohort	Chinese	1	January 1999 to December 2002	520	HBsAg + or HBeAg+	Not reported	Not reported	Matching^m^
Ryoo (37)	Korea	Retrospective cohort	English	1	April 1985 to June 1987	5,284	HBsAg+	8.48%	Not reported	No

a Part of pregnancies that received antiviral therapy.

b Conference abstract.

c Maternal age, educational level, insulin therapy during pregnancy, antiviral therapy during pregnancy, family history of diabetes, gestation at delivery, gravidity, parity, gestational weight gain, prepregnancy BMI, fasting plasma glucose during OGTT, 1-h plasma glucose during OGTT, and 2-h plasma glucose during OGTT.

d Maternal age, parity, abortion history, PTB history, CS history, high viral load, antiviral therapy, and abnormal liver function.

e Maternal age.

f Maternal age, parity, educational level, and job status.

g Sociodemographic variables, gestational characteristics, medical interventions, and gestational comorbidities.

h Maternal age, parity, socioeconomic, smoking, and alcohol intake status, and significant medical conditions.

i Maternal age, parity, year of delivery, and antenatal surveillance.

j Maternal age, parity, and body mass index.

k Maternal age, parity, and year of delivery.

l Maternal age, and year of delivery.

m Maternal age, parity, year of delivery, and gestational weeks.

HBV, hepatitis B virus; PPH, postpartum hemorrhage; HBsAg, hepatitis B surface antigen; HBeAg, hepatitis B e antigen.

Of these 21 studies, 14 reported the prevalence of HBV infection. Among them, 10 reported a prevalence rate higher than 3.61% (7, 10–12, 25–28, 30, 37), while four reported a prevalence rate lower than 3.61% (8, 29, 34, 35). Eleven studies adjusted for the effect of confounders when examining the association between HBV infection and PPH. Five studies were assessed using a multivariate analysis (10, 22, 23, 29, 30) and the remaining six using matching (24, 31, 33–36). All 21 studies scored in the 5–8 range, with 12 studies scoring 7 and above, indicating a relatively low risk of bias (Supplementary Table S1).

### 3.2 Association between maternal HBV infection and postpartum hemorrhage

**Figure 2:**
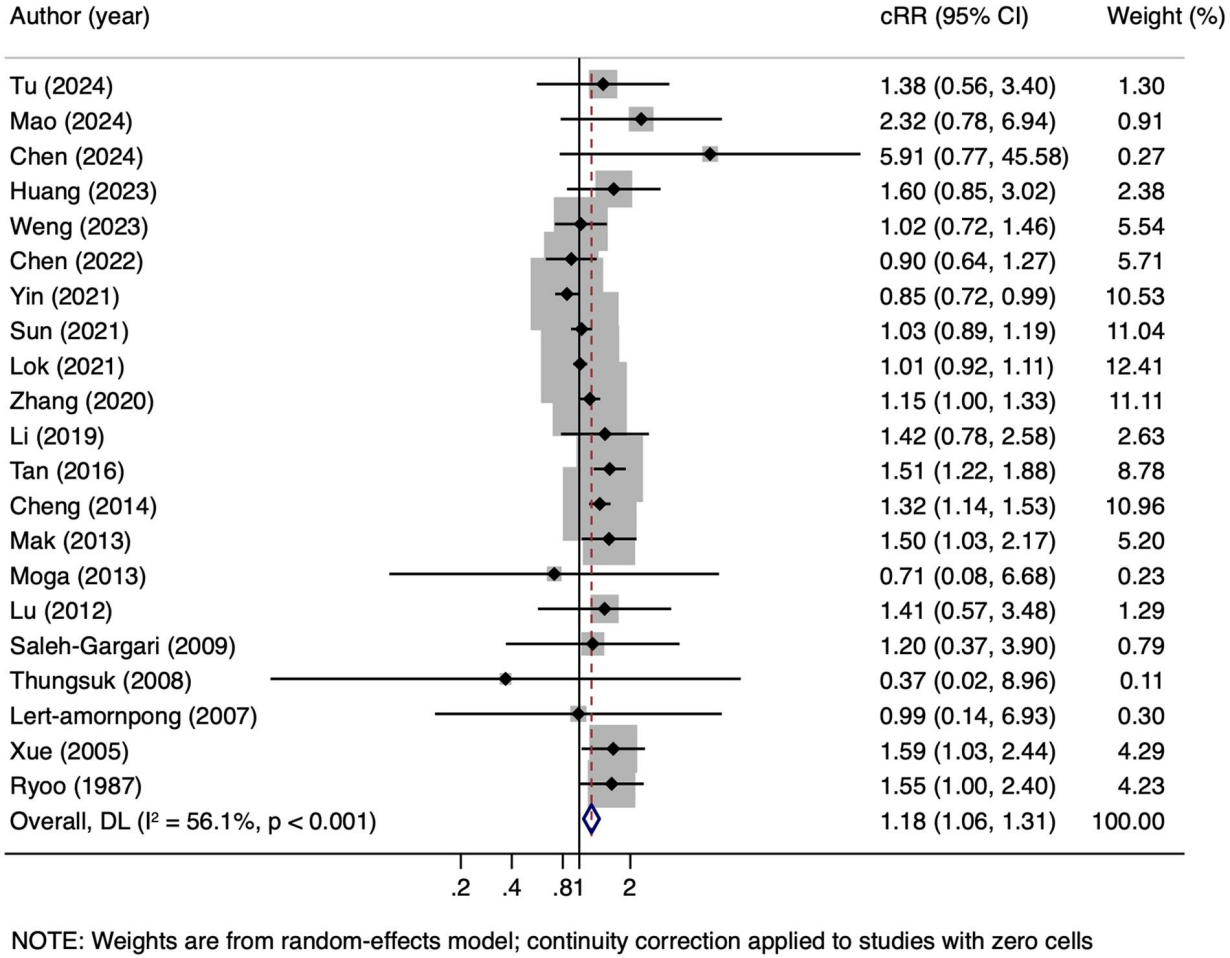
Unadjusted RR for risk of PPH in pregnant women with HBV infection. cRR, crude relative risk.

**Figure 3:**
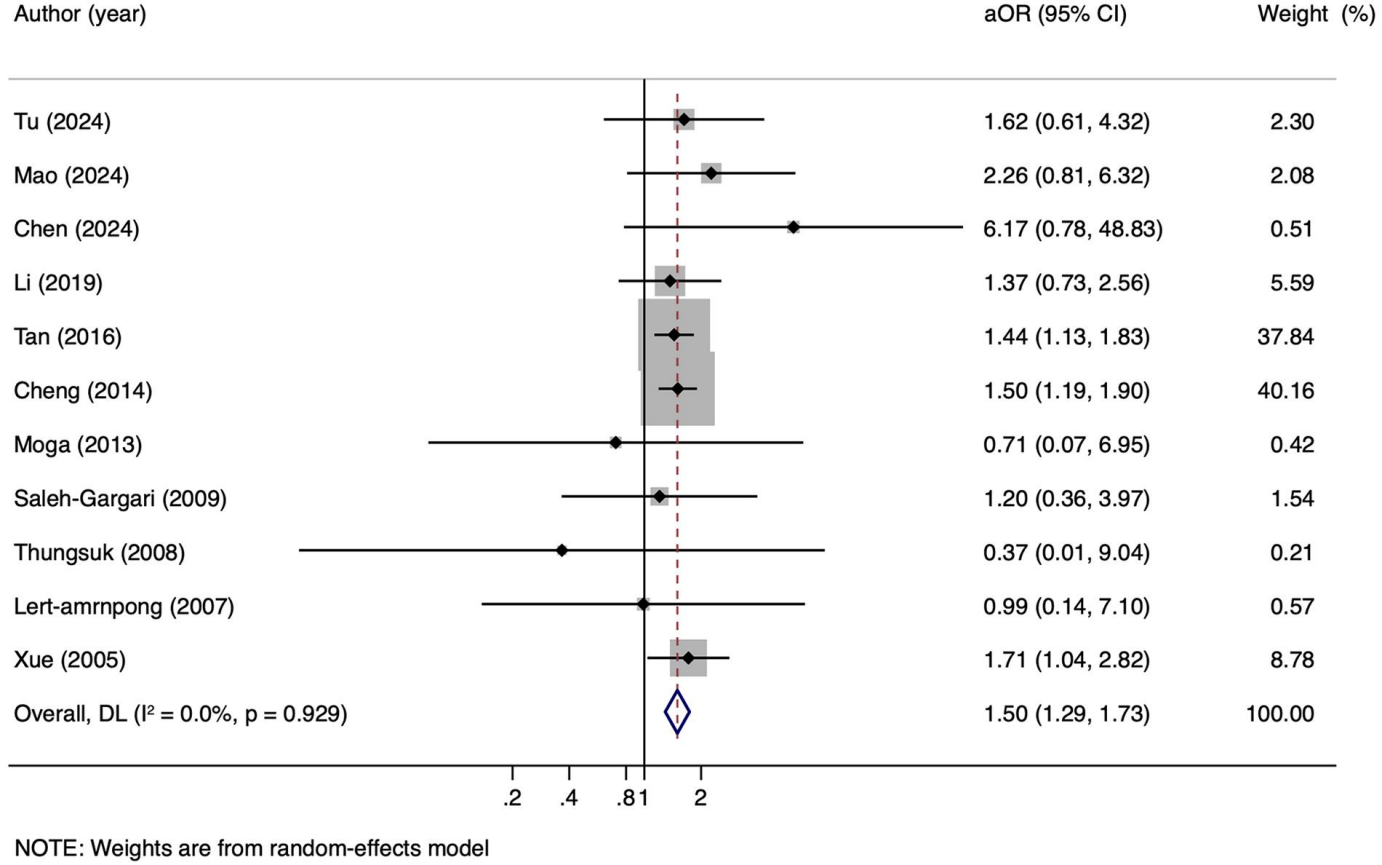
Adjusted OR for risk of PPH in pregnant women with HBV infection. aOR, adjusted odds ratio.

Twenty-one studies evaluated the association between maternal HBV infection and PPH, of which 11 provided adjusted results (10, 28, 29, 31, 33–36). The pooled results of the unadjusted data showed that maternal HBV infection was associated with an increased risk of PPH (1,426/31,069 vs. 22,556/348,713, cRR = 1.18, 95% CI: 1.06–1.31, *I^2^* = 56.1%) (Figure 2). Furthermore, the pooled results of the adjusted data indicated a similar risk of PPH (295/3,813 vs. 8,058/52,674, aOR = 1.50, 95% CI:1.29–1.73, *I^2^* = 0.0%) (Figure 3).

### 3.3 Subgroup and sensitivity analyses

According to the prespecified subgroup analyses, the majority of results indicated that maternal HBV infection was associated with an increased risk of PPH, which was consistent with the overall pooled results (Table 2). No statistically significant differences were found between sample size, study region, and prevalence of HBV infection in subgroup analyses in both unadjusted and adjusted pooled analyses (all *P*-values for interaction were greater than 0.05, Table 2).

**Table 2:** Results of subgroup analysis.

PPH	Unadjusted effect value (*n* = 21)				Adjusted effect values (*n* = 8)			
	No. of studies	cRR (95% CI)	*I*^2^ (%)	P for interaction	No. of studies	aOR (95% CI)	*I*^2^ (%)	*P* for interaction
Sample size								
<1,000	9	1.54 (1.12, 2.11)	0.00	0.09	8	1.67 (1.16, 2.40)	0.00	0.53
≥1,000	12	1.15 (1.02, 1.29)	70.50		3	1.46 (1.24, 1.72)	0.00	
Study region								
Asia	19	1.16 (1.04, 1.30)	53.71	0.19	9	1.50 (1.24, 1.82)	0.00	0.98
Non-Asia	2	1.32 (1.14, 1.52)	0.00		2	1.49 (1.18, 1.88)	0.00	
Prevalence of HBV infection^a^								
Lower (<3.61%)	5	1.16 (0.97, 1.39)	34.70	0.77	4	1.47 (1.17, 1.85)	0.00	0.86
Higher (≥3.61%)	16	1.20 (1.05, 1.37)	60.68		7	1.51 (1.24, 1.84)	0.00	

a Studies by Moga, Lu, Saleh-Gargari, and Xue, which did not explicitly report the prevalence, refer to the data reported by Schweitzer et al.

PPH, postpartum hemorrhage; cRR, crude relative risk; aOR, adjusted odds ratio; HBV, hepatitis B virus.

Sensitivity analyses results were mainly consistent with the primary results. After excluding studies with NOS scores less than 7, it was found that the pooled cRR was 1.16 (95% CI: 1.03–1.31) and the aOR was 1.48 (95% CI: 1.21–1.83) (Supplementary Table S2). After excluding studies published in non-English language, it was found that the pooled cRR was 1.16 (95% CI: 1.03–1.31) and the aOR was 1.49 (95% CI: 1.27–1.75). Upon excluding studies published as conference abstracts, it was found that the pooled cRR was 1.16 (95% CI: 1.04–1.30) and the aOR was1.49 (95% CI: 1.23–1.81). In addition, when only studies with HBsAg seropositivity during the first trimester were included, the pooled cRR was 1.10 (95% CI: 0.98–1.23) and the aOR was 1.40 (95% CI: 1.13–1.75). When only studies without antiviral therapy were included, the pooled cRR was 1.20 (95% CI: 1.06–1.36) and the aOR was 1.48 (95% CI: 1.27–1.72). Among the studies that specified the diagnostic criteria of PPH, the pooled cRR was 0.99 (95% CI: 0.89–1.11) and the aOR was 1.90 (95% CI: 0.94–3.86). In addition, trim-and-fill analyses showed that the pooled cRR was 1.16 (95% CI: 1.04–1.29) and the aOR was 1.50 (1.29–1.74) (Supplementary Table S2).

### 3.4 Publication bias

All funnel plots appeared symmetric, and Egger's test did not reveal significant publication bias either in the overall unadjusted pooled analyses (*P* = 0.12) or adjusted pooled analyses (*P* = 0.99) (Supplementary Figures S1 and S2).

## 4 Discussion

This study conducted a comprehensive systematic review and meta-analysis to investigate the association between maternal HBV infection and PPH. Our analysis indicated that maternal HBV infection was associated with an increased risk of PPH (pooled cRR = 1.18 and pooled aOR = 1.50). The results of several categories of sensitivity analyses were consistent with the primary results, indicating the robustness of the findings of this study.

Although this study investigated the association between maternal HBsAg+ and PPH, the impact of maternal HBeAg+ or HBV DNA levels on PPH remains largely unexplored. In general, HBeAg seropositivity or a high HBV DNA viral load, indicative of active HBV replication, may exert more severe effects on pregnancy outcomes than HBsAg seropositivity alone (38). For example, previous studies have reported more severe effects of HBeAg seropositivity or a high HBV DNA viral load on early preterm birth, neonatal asphyxia, GDM, and ICP (39, 40). For PPH, a study that included 201 HBV-infected pregnant women reported that the incidence of PPH in the maternal HBsAg+HBeAg+ group was higher than that in the maternal HBsAg+HBeAg- group (5.3% vs. 3.2%) (41). Similarly, the incidence of PPH was higher in women with HBV-DNA ≥34.2 copies·mL^−1^ than in women with HBV-DNA <34.2 copies mL^−1^ (6.0% vs. 3.0%) (41). However, another study of 260 pregnant women did not demonstrate a stronger effect for PPH in the HBsAg+HBeAg+ group (RR = 1.49) compared with that in the HBsAg+HBeAg- group (RR = 1.65) (36). Therefore, further studies are warranted to evaluate the impact of HBeAg serostatus and HBV DNA levels on PPH.

Although our study indicated that maternal HBV infection was a risk factor for PPH, the underlying mechanisms remain unclear. One potential pathway is that maternal HBV infection impairs liver function and modifies coagulation factors, resulting in impaired coagulation function in pregnant women during delivery, thereby increasing the risk of hemorrhage. For example, Dong et al. conducted a comparison of coagulation function between 398 HBV-infected and 400 HBV-non-infected pregnant women (42), which indicated that prothrombin time, activated partial thromboplastin time, thrombin time, and fibrinogen were significantly higher in HBV-infected pregnant women. In addition, HBV DNA levels were significantly correlated with activated partial thromboplastin time (*r* = 0.74, *P* < 0.05) (42). Considering the high prevalence of HBV infection among pregnant women, particularly in regions such as China, and the severity of PPH as an adverse pregnancy outcome, further research is imperative to elucidate the underlying mechanisms, which could significantly contribute to more effective prevention and reduction of PPH incidence.

This study clarifies the association between maternal HBV infection and PPH, offering multidisciplinary implications for clinical practice and public health. First, our findings provide the latest evidence to better understand the impact of HBV infection in specific populations, rather than just the general population. Second, our findings will encourage public health practitioners to advocate for and implement HBV vaccination and screening programs in women of childbearing age, thereby improving prenatal care services, particularly in regions with high HBV infection prevalence. Finally, further research and proactive public health measures are essential to improve the care of HBV-infected pregnant women and to elevate maternal and fetal health outcomes.

Our study has several strengths. First, based on currently available observational studies, this study is one of the few systematic reviews and meta-analyses to comprehensively evaluate the association between maternal HBV infection and PPH, providing clinically meaningful findings for clinical practice. Second, our search strategy included both Chinese and English databases. Given that China bears the highest burden of HBV infection in the world, the inclusion of Chinese databases significantly enhanced the comprehensiveness of our literature review. Third, we performed multiple subgroup analyses to investigate heterogeneity sources and conducted sensitivity analyses to examine the robustness of effect estimates.

This study also has a few limitations. First, in this study, some of the pooled results demonstrated heterogeneity, which could have been caused by differences in the inclusion criteria of the populations, basic characteristics of the populations, and definition of HBV infection or PPH across studies. Although we used random effects, the impact of heterogeneity on the pooled unadjusted results was not wholly eliminated. Second, among the included studies, only eight reported adjusted results, with several using matching methods for adjustment. The number of participants contributing adjusted results remained relatively limited. However, based on the available adjusted data, this study still indicated that maternal HBV infection increased the risk of PPH. Third, most studies did not record the diagnostic criteria for PPH, which would lead to diagnostic inconsistencies and thus reduce the precision of our results. Finally, the original studies included in this systematic review did not report detailed clinical information such as maternal liver disease severity, HBeAg serostatus, HBV DNA levels, and the presence of cirrhosis. Consequently, we were unable to perform analyses to determine the effects of this clinical information on PPH. Future research incorporating these clinical parameters is needed to strengthen the evidence.

This systematic review and meta-analysis evaluated the association between maternal HBV infection and PPH and found that maternal HBV infection might be associated with an increased risk of PPH. These findings contribute to improved prevention and management of PPH in clinical practice. Further studies are warranted to evaluate the impact of maternal HBeAg serostatus and HBV DNA levels on PPH.

## Data Availability

The original contributions presented in the study are included in the article/Supplementary Material; further inquiries can be directed to the corresponding authors.

## References

[1] WHO. Hepatitis B (2023) Available online at: https://www.who.int/news-room/fact-sheets/detail/hepatitis-b (Accessed July 18, 2023).

[2] WHO. Global Hepatitis Report (2017). Available online at: www.who.int/hepatitis/publications/global-hepatitis-report2017/en/ (Accessed June 24, 2017).

[3] Schweitzer A Horn J Mikolajczyk RT Krause G Ott JJ. Estimations of worldwide prevalence of chronic hepatitis B virus infection: a systematic review of data published between 1965 and 2013. Lancet. (2015) 386(10003):1546–55. 10.1016/s0140-6736(15)61412-x 26231459

[4] Ma X Sun D Li C Ying J Yan Y. Chronic hepatitis B virus infection and preterm labor(birth) in pregnant women—An updated systematic review and meta-analysis. J Med Virol. (2018) 90(1):93–100. 10.1002/jmv.24927 28851115

[5] Tan J Mao X Zhang G Wang W Pan T Liu X Hepatitis B surface antigen positivity during pregnancy and risk of gestational diabetes mellitus: a systematic review and meta-analysis. J Viral Hepat. (2018) 25(11):1372–83. 10.1111/jvh.12964 29968379

[6] Jiang R Wang T Yao Y Zhou F Huang X. Hepatitis B infection and intrahepatic cholestasis of pregnancy: a systematic review and meta-analysis. Medicine (Baltimore). (2020) 99(31):e21416. 10.1097/md.0000000000021416 32756142 PMC7402766

[7] Zhang Y Chen J Liao T Chen S Yan J Lin X. Maternal HBsAg carriers and pregnancy outcomes: a retrospective cohort analysis of 85,190 pregnancies. BMC Pregnancy Childbirth. (2020) 20(1):724. 10.1186/s12884-020-03257-4 33238912 PMC7687687

[8] Sun Q Lao TT Du M Xie M Sun Y Bai B Chronic maternal hepatitis B virus infection and pregnancy outcome-a single center study in Kunming, China. BMC Infect Dis. (2021) 21(1):253. 10.1186/s12879-021-05946-7 33691634 PMC7945294

[9] Watkins EJ Stem K. Postpartum hemorrhage. Jaapa. (2020) 33(4):29–33. 10.1097/01.Jaa.0000657164.11635.93 32224823

[10] Tan J Liu X Mao X Yu J Chen M Li Y HBsAg positivity during pregnancy and adverse maternal outcomes: a retrospective cohort analysis. J Viral Hepat. (2016) 23(10):812–9. 10.1111/jvh.12545 27167604

[11] Chen Y Ning W Wang X Chen Y Wu B Tao J. Maternal hepatitis B surface antigen carrier status and pregnancy outcome: a retrospective cohort study. Epidemiol Infect. (2022) 150:1–22. 10.1017/s0950268822000681 PMC9102056 35440355

[12] Weng M Wang J Yin J Ren W Wei C Yang W Effects of HBsAg carriers on pregnancy complications in pregnant women: a retrospective cohort study. Front Med (Lausanne). (2023) 10:1166530. 10.3389/fmed.2023.1166530 37293299 PMC10246503

[13] Stroup DF Berlin JA Morton SC Olkin I Williamson GD Rennie D Meta-analysis of observational studies in epidemiology: a proposal for reporting. Meta-analysis of observational studies in epidemiology (moose) group. JAMA. (2000) 283(15):2008–12. 10.1001/jama.283.15.2008 10789670

[14] A Guide to the Core Journal of China. Available online at: http://hxqk.lib.pku.edu.cn (Accessed April 18, 2024).

[15] Dahlke JD Mendez-Figueroa H Maggio L Hauspurg AK Sperling JD Chauhan SP Prevention and management of postpartum hemorrhage: a comparison of 4 national guidelines. Am J Obstet Gynecol. (2015) 213(1):76.e1–10. 10.1016/j.ajog.2015.02.023 25731692

[16] The American College of Obstetricians and Gynecologists. Practice Bulletin No. 183: postpartum hemorrhage. Obstet Gynecol. (2017) 130(4):e168–86. 10.1097/aog.0000000000002351 28937571

[17] Wells G Shea B O’Connell D Peterson J Welch V Losos M The Newcastle-Ottawa Scale (Nos) for Assessing the Quality of Nonrandomised Studies in Meta-Analyses (2011). Available online at: https://ohri.ca/en/who-we-are/core-facilities-and-platforms/ottawa-methods-centre/newcastle-ottawa-scale

[18] Higgins JP Thompson SG Deeks JJ Altman DG. Measuring inconsistency in meta-analyses. Br Med J. (2003) 327(7414):557–60. 10.1136/bmj.327.7414.557 12958120 PMC192859

[19] Altman DG Bland JM. Interaction revisited: the difference between two estimates. Br Med J. (2003) 326(7382):219. 10.1136/bmj.326.7382.219 12543843 PMC1125071

[20] Duval S Tweedie R. A nonparametric “trim and fill” method of accounting for publication bias in meta-analysis. J Am Stat Assoc. (2000) 95(449):89–98. 10.2307/2669529

[21] Egger M Davey Smith G Schneider M Minder C. Bias in meta-analysis detected by a simple, graphical test. Br Med J. (1997) 315(7109):629–34. 10.1136/bmj.315.7109.629 9310563 PMC2127453

[22] Tu Y Li Y Fan X Gui Z Dai J Fang Q Combined impact of hepatitis B virus and gestational diabetes Mellitus on ultrasound-measured fetal growth and adverse perinatal outcomes: a seven-year retrospective study. Diabetes Res Clin Pract. (2024) 207:111092. 10.1016/j.diabres.2024.111092 38219600

[23] Mao K Jiang P Cai W Lin Y Zhou Y Li D. Association of gestational hepatitis B virus infection and antiviral therapy with pregnancy outcomes: a retrospective study. Int J Gynecol Obstetrics. (2024) 166(1):115–25. 10.1002/ijgo.15716 38831742

[24] Chen C Wang ML Li WX Qi X Li Q Chen L. Hepatitis E virus infection increases the risk of obstetric complications and perinatal adverse outcomes in pregnant women with chronic hepatitis B virus infection. Eur Rev Med Pharmacol Sci. (2024) 28(5):1904–12. 10.26355/eurrev_202403_35604 38497873

[25] Huang W Wu X Yao Z Gu Y Lai X Meng L Investigating the relationship between hepatitis B virus infection and postpartum depression in Chinese women: a retrospective cohort study. Front Public Health. (2023) 11:1214151. 10.3389/fpubh.2023.1214151 38094232 PMC10716447

[26] Yin W Chen B Yang Y Li X Li R Xie J Association between maternal hepatitis B virus carrier and gestational diabetes mellitus: a retrospective cohort analysis. Virol J. (2021) 18(1):226. 10.1186/s12985-021-01691-0 34801053 PMC8605546

[27] Lok WY Kong CW To WWK. Prevalence of hepatitis B carrier status and its negative association with hypertensive disorders in pregnancy. Obstet Gynecol Int. (2021) 2021:9912743. 10.1155/2021/9912743 34691186 PMC8528634

[28] Li J Xu X Pei J Wang Y Liu Q Mao B. Study on maternal HBV carrier status and the pregnant outcomes [published in Chinese]. Mater Child Health Care China. (2019) 34(5):999–1002. 10.7620/zgfybj.j.issn.1001-4411.2019.05.10

[29] Cheng EH Witharana S Haque M. The impact of maternal chronic hepatitis B infection in obstetric outcomes. Hepatol Int. (2014) 8(1):S147–S8. 10.1007/s12072-014-9519-7

[30] Shui-Lam M Leung K-Y. Hepatitis B carriers in Hong Kong: prevalence and pregnancy outcomes. Hong Kong J Gynaecol, Obstet Midwifery. (2013) 13(1):67–73. 10.12809/hkjgom.13.1.142

[31] Moga M Anastasiu C Bâgiu N. Is maternal HBsAg carrier status associated with adverse pregnancy outcome? Ginecoeu. (2013) 9(1):8–10. 10.18643/gieu.2013.8

[32] Lu Y Chen Y Xiao X Liang X Li J Huang S Impact of maternal hepatitis B surface antigen carrier status on preterm delivery in southern China [published in Chinese]. J South Med Univ. (2012) 32(09):1369–72. 10.3969/j.issn.1673-4254.2012.09.036 22985586

[33] Saleh-Gargari S Hantoushzadeh S Zendehdel N Jamal A Aghdam H. The association of maternal HBsAg carrier status and perinatal outcome. Hepat Mon. (2009) 9(3):180–4.

[34] Thungsuk R. Maternal hepatitis B infection and pregnancy outcomes. J Prapokklao Hosp Clin Med Edu Center. (2008) 25(3):246–54.

[35] Lert-amornpong S Caengow S Chutaputti A. The association of pregnancy outcomes and HBsAg positive. Thai J Gastroenterol. (2007) 8(3):115–8.

[36] Xue Y Yu M Wei M. Observation on maternal infant outcome in pregnancy with hepatitis B virus infection [published in Chinese]. Shanghai Med J. (2005) 9:747–9.

[37] Ryoo YG Chang YH Choi GS Jeong WJ Kim JW Joung NK Hepatitis B viral markers in pregnant women and newborn infants in Korea. Korean J Intern Med. (1987) 2(2):258–68. 10.3904/kjim.1987.2.2.258 3154838 PMC4534940

[38] Sirilert S Tongsong T. Hepatitis B virus infection in pregnancy: immunological response, natural course and pregnancy outcomes. J Clin Med. (2021) 10(13):2926. 10.3390/jcm10132926 34210105 PMC8267880

[39] Liu J Zhang S Liu M Wang Q Shen H Zhang Y. Maternal Pre-pregnancy infection with hepatitis B virus and the risk of preterm birth: a population-based cohort study. Lancet Glob Health. (2017) 5(6):e624–32. 10.1016/s2214-109x(17)30142-0 28495266

[40] Wu K Wang H Li S Zhang H Zhu B. Maternal hepatitis B infection status and adverse pregnancy outcomes: a retrospective cohort analysis. Arch Gynecol Obstet. (2020) 302(3):595–602. 10.1007/s00404-020-05630-2 32705338

[41] Li Y Zou M Zhang L Ming F. Clinical study on the correlation between changes in liver function indicators in pregnancy complicated with chronic hepatitis B and maternal and infant complications [published in Chinese]. J Nantong Uni (Medical Sciences). (2022) 42(4):380–4. 10.16424/j.cnki.cn32-1807/r.2022.04.022

[42] Dong W. Significance of pre-S1 and blood coagulation index detection in HBV positive pregnant women [published in Chinese]. Chin J Mod Drug Appl. (2021) 15(10):54–6. 10.14164/j.cnki.cn11-5581/r.2021.10.019

