# Supplementary material for: Effect of Hepatitis B virus infection during pregnancy on the risk of postpartum hemorrhage: a systematic review and meta-analysis
Source: Front Glob Womens Health. 2026 Jan 9;6:1596520. doi: 10.3389/fgwh.2025.1596520 (PMC12827722; doi:10.3389/fgwh.2025.1596520)
Supplement: Supplementary file 1 [file Table1.docx]

**Table S1.** Risk of bias of included studies

| **Study** | **Selection** | | | | **Comparability** | **Outcome** | | | **Overall score** |
| --- | --- | --- | --- | --- | --- | --- | --- | --- | --- |
|  | **Representative-ness of the exposed cohort** | **Selection of non-exposed cohort** | **Ascertainment of exposure** | **Outcome of interest was not present at start** | **Controls for important risk factors** | **Assessment of outcome** | **Follow-up long enough** | **Adequacy of follow up of cohorts** |  |
| Tu (2024) ^22^ | - | * | * | * | - | * | * | * | 6 |
| Mao (2024) ^23^ | - | * | * | * | * | * | * | * | 7 |
| Chen (2024) ^24^ | - | * | * | * | * | - | * | * | 6 |
| Huang (2023) ^25^ | - | * | * | * | * | * | * | * | 7 |
| Weng (2023) ^12^ | - | * | * | * | * | - | * | * | 6 |
| Chen (2022) ^11^ | - | * | * | * | * | * | * | * | 7 |
| Yin (2021) ^26^ | - | * | * | * | - | * | * | * | 6 |
| Sun (2021) ^8^ | - | * | * | * | ** | * | * | * | 8 |
| Lok (2021) ^27^ | - | * | * | * | ** | * | * | * | 8 |
| Zhang (2020) ^7^ | - | * | * | * | ** | - | * | * | 7 |
| Li (2019) ^28^ | - | * | * | * | * | - | * | * | 6 |
| Tan (2016) ^10^ | * | * | * | * | * | - | * | * | 7 |
| Cheng (2014) ^29^ | - | * | * | * | - | - | * | * | 5 |
| Mak (2013) ^30^ | - | * | * | * | * | - | * | * | 6 |
| Moga (2013) ^31^ | - | * | * | * | ** | - | * | * | 7 |
| Lu (2012) ^32^ | - | * | * | * | ** | - | * | * | 7 |
| Saleh-Gargari (2009) ^33^ | - | * | * | * | ** | - | * | * | 7 |
| Thungsuk (2008) ^34^ | - | * | * | * | ** | - | * | * | 7 |
| Lert-amornpong (2007) ^35^ | - | * | * | * | * | - | * | * | 6 |
| Xue (2005) ^36^ | - | * | * | * | ** | - | * | * | 7 |
| Ryoo (1987) ^37^ | - | * | * | * | - | - | * | * | 5 |
