# Supplementary material for: Effect of Hepatitis B virus infection during pregnancy on the risk of postpartum hemorrhage: a systematic review and meta-analysis
Source: Front Glob Womens Health. 2026 Jan 9;6:1596520. doi: 10.3389/fgwh.2025.1596520 (PMC12827722; doi:10.3389/fgwh.2025.1596520)
Supplement: Supplementary file 2 [file Table2.docx]

**Table S2.** Results of sensitivity analyses

| **PPH** | **No. of studies** | **cRR/aOR (95% CI)** | ***I^2^*(%)** |
| --- | --- | --- | --- |
| **Omitting articles with NOS<7 scores** | | | |
| cRR | 12 | 1.16 (1.03, 1.31) | 46.23 |
| aOR | 6 | 1.48 (1.21, 1.83) | 0.00 |
| **Omitting articles published in non-English language** | | | |
| cRR | 17 | 1.16 (1.03, 1.31) | 61.38 |
| aOR | 8 | 1.49 (1.27, 1.75) | 0.00 |
| **Omitting articles published in conference abstract** | | | |
| cRR | 20 | 1.16 (1.04, 1.30) | 51.30 |
| aOR | 10 | 1.49 (1.23, 1.81) | 0.00 |
| **Omitting articles involved HBV serologic testing not during first trimester** | | | |
| cRR | 13 | 1.10 (0.98, 1.23) | 56.84 |
| aOR | 5 | 1.40 (1.13, 1.75) | 0.00 |
| **Omitting articles involved part of pregnancies that received antiviral therapy** | | | |
| cRR | 18 | 1.20 (1.06, 1.36) | 55.37 |
| aOR | 9 | 1.48 (1.27, 1.72) | 0.00 |
| **Omitted articles did not report the diagnostic criteria of PPH** | | | |
| cRR | 7 | 0.99 (0.89, 1.11) | 37.36 |
| aOR | 2 | 1.90 (0.94, 3.86) | 0.00 |
| **Imputed potentially missing studies** | | | |
| cRR | 24 | 1.16 (1.04, 1.29) | - |
| aOR | 12 | 1.50 (1.29, 1.74) | - |

Note:

PPH, postpartum hemorrhage; cRR, crude relative risk; aOR, adjusted odds ratio; NOS, Newcastle-Ottawa Scale; HBV, hepatitis B virus
